# Murine Model for Parkinson's Disease: from 6-OH Dopamine Lesion to Behavioral Test

**DOI:** 10.3791/1376

**Published:** 2010-01-15

**Authors:** Fabio S.L. da Conceição, Stacie Ngo-Abdalla, Jean-Christophe Houzel, Stevens K. Rehen

**Affiliations:** Instituto de Ciências Biomédicas, Universidade Federal do Rio de Janeiro, Brasil

## Abstract

Parkinson's disease (PD) affects at least 6.5 million people worldwide, irrespective of gender, social, ethnic, economic, or geographic boundaries. Key symptoms, such as tremor, rigidity and bradikinesia, develop when about 3/4 of dopaminergic cells are lost in the substantia nigra, and fail to provide for the smooth, coordinated regulation of striatal motor circuits. Depression and hallucinations are common, and dementia eventually occurs in 20% of patients. At this time, there is no treatment to delay or stop the progression of PD. Rather, the medications currently available aim more towards the alleviation of these symptoms. New surgical strategies may reversibly switch on the functionally damaged circuits through the electrical stimulation of deep brain structures, but although deep brain stimulation is a major advance, it is not suitable for all patients. It remains therefore necessary to test new cell therapy approaches in preclinical models.

Selective neurotoxic disruption of dopaminergic pathways can be reproduced by injection of 6-hydroxydopamine (6-OHDA) or MPTP (1-methyl-4-phenyl-1,2,3,6-tertahydropyridine) whereas depleting drugs and oxidative-damaging chemicals may also reproduce specific features of PD in rodents. Unlike MPTP, 6-OHDA lesions cause massive irreversible neuronal loss, and can be uni- or bilateral. The 6-OHDA lesion model is reliable, leads to robust motor deficits, and is the most widely used after 40 years of research in rats1. As interactions between grafted cells and host can now be studied more thoroughly in mice rather than in rats, the model has been transposed to mice^2,3^, where it has been recently characterized^4^.

In this video, we demonstrate how to lesion the left nigro-striatal pathway of anesthetized mice by slowly delivering 2.0 μL of 6-OHDA through a stereotaxically inserted micro-syringe needle. The loss of dopaminergic input occurs within days, and the functional impairments can be monitored over post-operative weeks and months by rating animal rotations induced by dopaminergic agents^5^. Here, we show full-body contralateral rotations occurring 10 minutes after a single subcutaneous administration of apomorphine, measured one month after the lesion. Outcomes and drawbacks are discussed below.

**Figure:**
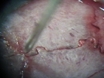


## Protocol

All chemicals stored in solid form, such as 6-OHDA and apomorphine, were diluted in sterile injection water and filtered inside a laminar flow hood, to avoid contamination. In addition, neurotoxins and neuroactive substances were stored, prepared, handled and disposed according to regulations set by international guidelines^6^ and the local Biosafety Committee. 

### 1 - Anesthesia and surgery

Adult male Swiss mice (25-30g) are premedicated with Atropine (0.2 mg/Kg, s.c.) to reduce vagal tone and then anesthetized with an intraperitoneal injection of Ketamine (90-120 mg/Kg) and Xylazine (10 mg/Kg). The animal is placed on an electronically controlled heating pad (Insight) to ensure body temperature is maintained at 37°C, as induction of sedation takes some minutes. The anesthesia level is checked throughout the procedure by testing the absence of withdrawal reflex. Anesthetic dosage should be ajusted for each strain/colony. Depending on the animal and duration of the surgery, additional doses of Ketamine may be necessary. After that, saline (NaCl 0,9%) is applied on both eyes to protect the corneas from drying out and test the absence of the blink reflex in this first moment. The fur over the entire head is shaved with a trimmer to ensure the minimum contact with unsterile areas. Local anesthetic (Xylocaine) is applied to the ear bars to prevent discomfort. Only once withdrawal and blink reflex have vanished, indicating the level of anesthesia is deep enough for surgery (stage 3), the animal is positioned on the stereotaxic frame using mouth-piece and ear bars specially designed for this species (Insight). Once positionated, an ophthalmologic ointment (MARCA) is applied to protect the animal eyes throughout all surgery. Only after be sure that the eyes are correctly protected, the procedure continue with the disinfection of the skin of the surgical region by repeating three applications of povidone iodine followed by 70% Ethanol, using sterile cottons swabs After, proper placement of the ear bars is ensured by testing for sideways movements of the head (the head must not move to ensure that the bars are securely fixed). Proper dorsoventral inclination of the head will be verified later when skull landmarks are revealed. A sagittal incision (1.5 cm) is made with a sterile scalpel (#15 blade). The periosteum over the area of interest is gently scraped away with a new blade, and the bone is wiped clean with sterile gaze and cotton swabs, to uncover skull landmarks: bregma is defined as the point of intersection of sagittal and coronal sutures, while lambda is the point of intersection of the sagittal suture and the best fit line passing through the left and right portions of the lambdoid suture. The tip of a vertically aligned reference needle is first zeroed at the bregma, under examination through the surgical microscope, and then moved as to touch the lambda point. If the head is properly positioned, lambda should be at the same dorso-ventral level as bregma, and both landmarks should be distant by 4.2 mm along the rostro-caudal axis. If not, the ears bars and nosepiece should be loosen and the head delicately tilted around the interaural axis, in order to achieve the desired position, which defines the "flat skull" reference for stereotaxical measures. The tip of the reference needle is then positioned at the desired coordinates: anterior-posterior (AP +0.5 mm) and lateral (L -2.0 mm, left). The location is marked on the skull and a small (1.2 mm diameter) hole is opened with a sterilized drill bit, using intermittent action to prevent heat the area. Bone fragments are carefully removed with a dental curette and washed with sterile warm 0.9% NaCl. The sterilized 5 μl Hamilton syringe (26s gauge; 0.47 mm outer diameter) is loaded with the 6-OHDA solution (or vehicle for control animals) and vertically aligned in the stereotaxic apparatus. The tip of the needle (type 2 or 12° bevel) is then inserted into the opened hole, until touch the pial surface, to determinate the reference point of the dorso-ventral coordinate. Once determinate this coordinate, the needle is slowly lowered to reach the coordinates of the striatum (AP: +0.5; L: -2.0 and DV: -3.0 mm, below pial surface^7^). The 6-OHDA solution (10 μg 6-OHDA in 0.9% NaCl with 0.02% ascorbic acid), which was prepared and filtered inside a laminar flow hood, and must be protected from light by aluminum foil throughout the procedure, is then injected at a flow rate of 0.1μL/min. For control animals, an equal volume of vehicle (0.02% ascorbic acid and 0.9% NaCl in sterile water) is injected. Once the entire 2.0 μL has been injected, the syringe is kept in place for 5 min before being very slowly retracted from the brain, in no less than in 5 minutes. The skull is cleaned and the incision is closed using a #6-0 nylon suture (Shalon). To take a extra care against infection, a local antibiotic ointment, such as neomycin, may be applied to the sutured skin. Animals are removed from the frame, given 0.5 ml of saline (sc.) to prevent dehydration, and kept warm and under close observation until full recovery. They are then housed with food and water ad libitum. Analgesic is added to the drinking water bottle for the first two days after surgery (given an average daily water consumption of 5.5 ml, and a recommended dosage of 0.03 mg Ibuprofen/g of body weight/day; add 0,82 mL of 20 mg/mL Ibuprofen-Abbott suspension to 100 mL drinking water). Mice usually recover promptly from the procedure, wandering and reaching for water food pellets easily. In any case of apparent discomfort, or any sign of infection, animals should be euthanized according to local guidelines.

### 2 - Behavioral test

Although motor impairment can be observed after a week, a post-lesion interval of one month is allowed before reliable quantification with the rotation test. The animal receives a subcutaneous injection of DA agonist apomorphine (0.5 mg/Kg), and is placed in an opaque cylinder of 30 cm diameter, placed 45 cm below the recording camera. It is important to remove any residues and odors from previously tested animals before each test. After a 5 min habituation period, movements are recorded over a 5 min timeframe. Both contra- and ipsilateral full-body rotation are measured and compared with the control group (vehicle injected). If the procedure was correctly performed, control animals will not exhibit any rotation, whereas 6-OHDA lesioned animals will start turning to the contralateral side, since the agonist predominantly activates the supersensitive denervated striatum on the lesioned side. Animals scoring over 7 rpm (displaying at least 7 full-body contralateral rotations per minute) are considered as successfully lesioned. The animal can be safely returned to their housing 30 min after the test, which may be repeated weekly. In our hands, less than 5% of the lesioned mice displayed suboptimal rotation rates.

## Disclosures

Experiments were performed in accordance with the international Guidelines for the Care and Use of Mammals in Neuroscience and Behavior Research<sup>6</sup>, after approval by the Institutional Animal Care and Use Committee (Comiss&atilde;o de Avaliaç&atilde;o de Uso de Animais em Pesquisa: UFRJ/CAUAP protocol #DAHEICB 027). Adequate measures were taken to minimize the pain and discomfort of the experimental animals.

## Discussion

Extensive unilateral lesion of the nigro-striatal pathway can be achieved reliably in mice by a single stereotaxic injection of 6-OHDA into the striatum. The extent of the lesion can be verified post-mortem by immunohistochemistry for Tyrosine Hydroxylase (which catalyses the rate limiting stage of the synthesis of DA, i.e. the conversion of L-Tyrosine to dihydroxyphenylalanine), as illustrated in the video.

In vivo, contralateral rotations induced by apomorphine are a better predictor of maximal striatal lesion than amphetamine-induced ipsilateral rotation^8^. Thus, functional consequences of the lesion can be monitored over several months by a straightforward measure of apomorphine-induced contralateral rotations.

The success rate is about 95%. It is significantly higher than what is usually reported for rats (50-70%) in which extensive dopaminergic depletion requires multiples injections of the neurotoxin and increases the mortality rate. In mice, experimental conditions should be standardized by selecting animals of matching weight, and only using freshly prepared 6-OHDA solution. Although other groups may use different concentration and/or volumes of neurotoxin, we have found that 2 ml of a 5 mg/ml saline solution of 6-OHDA (containing 0.02% ascorbic acid to prevent oxidation) were the most efficient for maximal lesion. Alternatively, submaximal injections could be useful to mimic the progressive degeneration of dopaminergic terminals that occurs in humans, especially in juvenile and early-onset forms of PD^9^.

MPTP lesions may lead to spontaneous recovery, and additionally is sensitive to gender, age, and strain. A recent study indicates that this is not the case for 6-OHDA lesions in mice, which yield massive and long lasting reductions in the residual DA content within the striatum, as well as in the number of TH positive cells in the substantia nigra in all mice tested^10^.

At the present time, the 6-hydroxydopamine lesion model of Parkinson - which has been extensively used for 40 years in rats -, can be reliably transposed to mice. We hope that this video will be helpful to other groups, and thereby reduce the number of experiments and animals needed for preclinical research on new cellular therapies for PD^11^.
